# Patterns in Cancer Incidence Among People Younger Than 50 Years in the US, 2010 to 2019

**DOI:** 10.1001/jamanetworkopen.2023.28171

**Published:** 2023-08-16

**Authors:** Benjamin Koh, Darren Jun Hao Tan, Cheng Han Ng, Clarissa Elysia Fu, Wen Hui Lim, Rebecca Wenling Zeng, Jie Ning Yong, Jia Hong Koh, Nicholas Syn, Wang Meng, Karn Wijarnpreecha, Ken Liu, Choon Seng Chong, Mark Muthiah, Hung N. Luu, Arndt Vogel, Siddharth Singh, Khay Guan Yeoh, Rohit Loomba, Daniel Q. Huang

**Affiliations:** 1 Yong Loo Lin School of Medicine, National University of Singapore, Singapore; 2 Division of Gastroenterology and Hepatology, Department of Medicine, National University Hospital, Singapore; 3 National University Centre for Organ Transplantation, National University Health System, Singapore; 4 Division of Hepatobiliary and Pancreatic Surgery, Department of Surgery, University Surgical Cluster, National University Hospital, Singapore; 5 Cancer Science Institute of Singapore, National University of Singapore, Singapore; 6 Department of Medicine, Division of Gastroenterology and Hepatology, University of Arizona College of Medicine, Phoenix; 7 AW Morrow Gastroenterology and Liver Centre, Royal Prince Alfred Hospital, Sydney, Australia; 8 Ark Surgical Practice, Mount Elizabeth Hospital, Singapore, Singapore; 9 Division of Cancer Control and Population Sciences, UPMC Hillman Cancer Center, Pittsburgh, Pennsylvania; 10 Department of Epidemiology, School of Public Health, University of Pittsburgh, Pittsburgh, Pennsylvania; 11 Department of Gastroenterology, Hepatology and Endocrinology, Hannover Medical School, Hannover, Germany; 12 Division of Gastroenterology, University of California at San Diego, La Jolla

## Abstract

**Importance:**

Emerging data suggest that the incidence of early-onset cancers, defined as cancers diagnosed in people younger than 50 years, is increasing, but updated data are limited.

**Objective:**

To characterize the patterns in the incidence of early-onset cancers in the US from 2010 to 2019 and provide granular data on the cancers with the fastest-growing incidence rates.

**Design, Setting, and Participants:**

This population-based cohort study analyzed data from 17 National Cancer Institute Surveillance, Epidemiology, and End Results registries from January 1, 2010, to December 31, 2019. Age-standardized incidence rates per 100 000 people were extracted for early-onset cancers, with rates age adjusted to the US standard population. A total of 562 145 patients with early-onset cancer between 2010 and 2019 were identified and included. Data were analyzed from October 16, 2022, to May 23, 2023.

**Main Outcomes and Measures:**

Primary outcomes were incidence rates and descriptive epidemiological data for people younger than 50 years with cancer. The annual percentage change (APC) of the age-standardized incidence rate was estimated using the Joinpoint regression program.

**Results:**

Among 562 145 patients (324 138 [57.7%] aged 40-49 years; 351 120 [62.5%] female) with early-onset cancer, 4565 (0.8%) were American Indian or Alaska Native, 54 876 (9.8%) were Asian or Pacific Islander, 61 048 (10.9%) were Black, 118 099 (21.0%) were Hispanic, 314 610 (56.0%) were White, and 8947 (1.6%) were of unknown race and/or ethnicity. From 2010 to 2019, the age-standardized incidence rate of early-onset cancers increased overall (APC, 0.28%; 95% CI, 0.09%-0.47%; *P* = .01) and in female individuals (APC, 0.67%; 95% CI, 0.39%-0.94%; *P* = .001) but decreased in male individuals (APC, −0.37%; 95% CI, −0.51% to −0.22%; *P* < .001). In contrast, the age-standardized incidence rate of cancers in individuals aged 50 years and older decreased over the study period (APC, −0.87%; 95% CI, −1.06% to −0.67%; *P* < .001). In 2019, the highest number of incident cases of early-onset cancer were in the breast (n = 12 649). From 2010 to 2019, gastrointestinal cancers had the fastest-growing incidence rates among all early-onset cancer groups (APC, 2.16%; 95% CI, 1.66%-2.67%; *P* < .001). Among gastrointestinal cancers, those with the fastest-growing incidence rates were in the appendix (APC, 15.61%; 95% CI, 9.21%-22.38%; *P* < .001), intrahepatic bile duct (APC, 8.12%; 95% CI, 4.94%-11.39%; *P* < .001), and pancreas (APC, 2.53%; 95% CI, 1.69%-3.38%; *P* < .001).

**Conclusions and Relevance:**

In this cohort study, the incidence rates of early-onset cancer increased from 2010 to 2019. Although breast cancer had the highest number of incident cases, gastrointestinal cancers had the fastest-growing incidence rates among all early-onset cancers. These data may be useful for the development of surveillance strategies and funding priorities.

## Key Points

**Question:**

What are the patterns in the incidence of cancers in people younger than 50 years (ie, early-onset cancers)?

**Findings:**

In this cohort study of 562 145 people with early-onset cancer in the US from 2010 to 2019, the incidence rates of early-onset cancers increased substantially over the study period. Gastrointestinal cancers had the fastest-growing incidence rates among all early-onset cancers.

**Meaning:**

These data may be useful for the development of surveillance strategies and funding priorities.

## Teaser

This cohort study uses data from Surveillance, Epidemiology, and End Results registries to assess patterns in the incidence of early-onset cancers from 2010 to 2019 in the US.

## Introduction

Cancer has traditionally been considered a disease of older individuals (defined as adults 50 years and older),^1^ but recent data suggest a marked increase in the incidence of cancer of various organs among patients younger than 50 years, collectively known as early-onset cancer.^2,3^ These cancers affect a variety of organ systems, including the breast, colon and/or rectum, pancreas, head and neck, kidney, and reproductive organs.^2,4,5,6,7,8^ The increase in early-onset cancers is likely associated with the increasing incidence of obesity as well as changes in environmental exposures, such as smoke and gasoline,^9^ sleep patterns, physical activity, microbiota, and transient exposure to carcinogenic compounds.^10,11,12,13,14^

Early-onset cancer is associated with substantial mortality and morbidity.^10,15,16,17,18^ Recent efforts have evaluated the incidence patterns of specific types of early-onset cancers.^19,20,21,22,23^ However, an updated comprehensive overview of recent patterns of early-onset cancer in the US that is not limited to specific organ systems has not been reported.^24^ This study used population-based data from the National Cancer Institute Surveillance, Epidemiology, and End Results (SEER) program to characterize temporal patterns in the incidence of early-onset cancers in the US from 2010 to 2019. The primary objective was to characterize the temporal patterns in early-onset cancer overall and by organ system. The secondary objective was to assess temporal patterns in early-onset cancer stratified by sex, age group, and race and ethnicity.

## Methods

This cohort study was conducted in accordance with the Declaration of Helsinki.^25^ The study was exempt from institutional review board review because no confidential patient information was involved per the 2018 Revised Common Rule (45 CFR §46).^26^ This study followed the Strengthening the Reporting of Observational Studies in Epidemiology (STROBE) reporting guideline for cohort studies.

### Data Sources

The National Cancer Institute SEER program collects population-based data on cancer incidence in the US. The SEER database is a network of tumor registries from various geographically distinct regions within the country and contains representative data for the racial and ethnic diversity present within the US.^27^ The data in the SEER registry are sourced annually from the US Census Bureau, and the registry is estimated to cover approximately 26.5% of the population in the US.^28^ In this study, data from 17 SEER registries (hereinafter, SEER 17 database) were used to estimate the number of incident cancer cases from January 1, 2010, to December 31, 2019, as well as the cross-sectional incidence rates and the 10-year mean annual percentage change (APC) in incidence rates. Race and ethnicity categories were determined per the SEER coding manual, which was predominantly based on self-reported data. Data were analyzed from October 16, 2022, to May 23, 2023.

### Statistical Analysis

Demographic data were obtained using SEER*Stat software, version 8.4.0.1 (National Cancer Institute),^29^ from incident cases of cancer diagnosed between 2010 and 2019 and collected from the SEER 17 database. Population data were segregated based on age, race and ethnicity, and sex available within SEER*Stat. Incidence rates were calculated using SEER*Stat and were age standardized to the 2000 US standard population.^30^ Tiwari et al^31^ modification was applied for more efficient calculation of CIs for rates and rate ratios. All cases of cancer were classified according to the World Health Organization 2008 definitions, which were based on the *International Classification of Diseases for Oncology*, third edition.^32^ Cancers were grouped according to organ systems (eTable 1 in Supplement 1). The APC of incidence rates was quantified using the Joinpoint regression program, version 5.0.2 (National Cancer Institute).^33^ Temporal patterns from 2010 to 2019 were evaluated to identify changes that may have occurred over the study period. Rates and APCs across the strata of sex (male or female), race and ethnicity (Hispanic, non-Hispanic American Indian or Alaska Native, non-Hispanic Asian or Pacific Islander, non-Hispanic Black, non-Hispanic White, or unknown race and/or ethnicity), and age group (0-19 years, 20-29 years, 30-39 years, or 40-49 years) were evaluated to identify populations at risk of developing cancer. Analysis of American Indian or Alaska Native populations was restricted to areas with health care services provided by the Indian Health Services for improved accuracy of racial classification.^34^ As a result, 960 persons from outside of purchased or referred care delivery areas were excluded from the analysis. The Monte Carlo permutation method was used to test for significance,^33^ and 2-sided *P* < .05 was considered statistically significant.

## Results

### Characteristics of the Study Population

Between 2010 and 2019, a total of 562 145 patients with early-onset cancer (324 138 [57.7%] aged 40-49 years; 351 120 [62.5%] female and 211 025 [37.5%] male) were identified over 2 102 085 738 person-years of observation. A total of 4565 patients (0.8%) were American Indian or Alaska Native, 54 876 (9.8%) were Asian or Pacific Islander, 61 048 (10.9%) were Black, 118 099 (21.0%) were Hispanic, 314 610 (56.0%) were White, and 8947 (1.6%) were of unknown race and/or ethnicity (eTable 2 in Supplement 1).

### Patterns in the Overall Incidence of Early-Onset Cancer From 2010 to 2019

**Figure 1.**
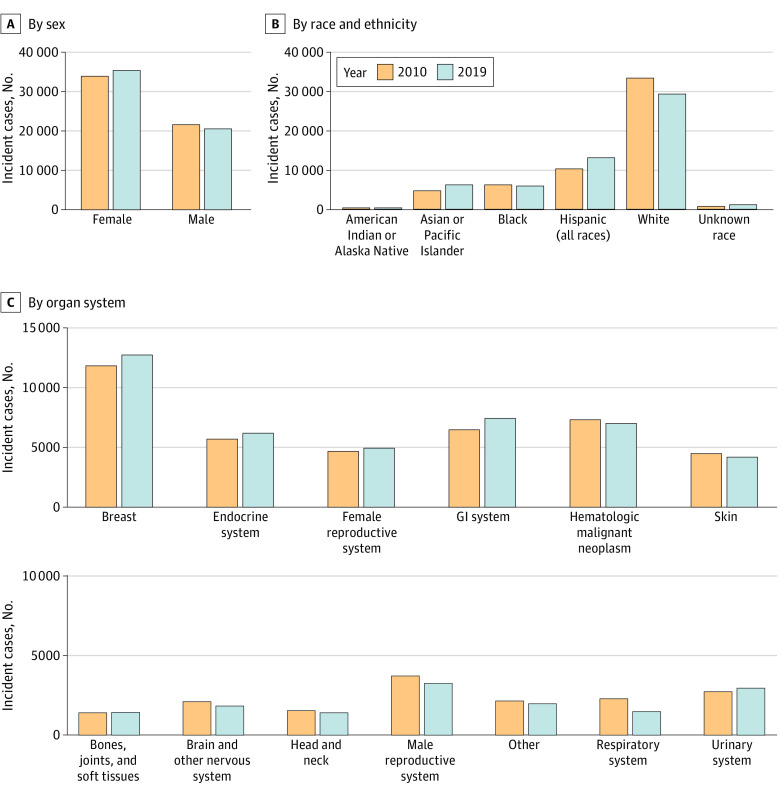
Incident Cases of Early-Onset Cancer in 2010 and 2019 GI indicates gastrointestinal.

The number of incident early-onset cancers was 56 051 in 2010 and 56 468 in 2019, representing an increase of 0.74% during the study period. The number of incident early-onset cancers in male individuals was 21 818 in 2010 and 20 747 in 2019, representing a decrease of 4.91%; the number of incident early-onset cancers in female individuals was 34 233 in 2010 and 35 721 in 2019, representing an increase of 4.35% (Figure 1A). By race and ethnicity, the number of incident early-onset cancers in 2010 was 351 among American Indian or Alaska Native people, 4723 among Asian or Pacific Islander people, 6245 among Black people, 10 326 among Hispanic people, and 33 578 among White people; the number of incident early-onset cancers in 2019 was 359 among American Indian or Alaska Native people, 6246 among Asian or Pacific Islander people, 5953 among Black people, 13 177 among Hispanic people, and 29 481 among White people (Figure 1B). These values represented an increase of 2.28% among American Indian or Alaska Native people, 32.25% among Asian or Pacific Islander people, and 27.61% among Hispanic people but a decrease of 4.68% among Black people and 12.20% among White people. When stratified by specific age groups, the number of incident early-onset cancers in 2019 was 3983 among individuals aged 0 to 19 years, 5899 among individuals aged 20 to 29 years, 14 762 among individuals aged 30 to 39 years, and 31 824 among individuals aged 40 to 49 years.

**Figure 2.**
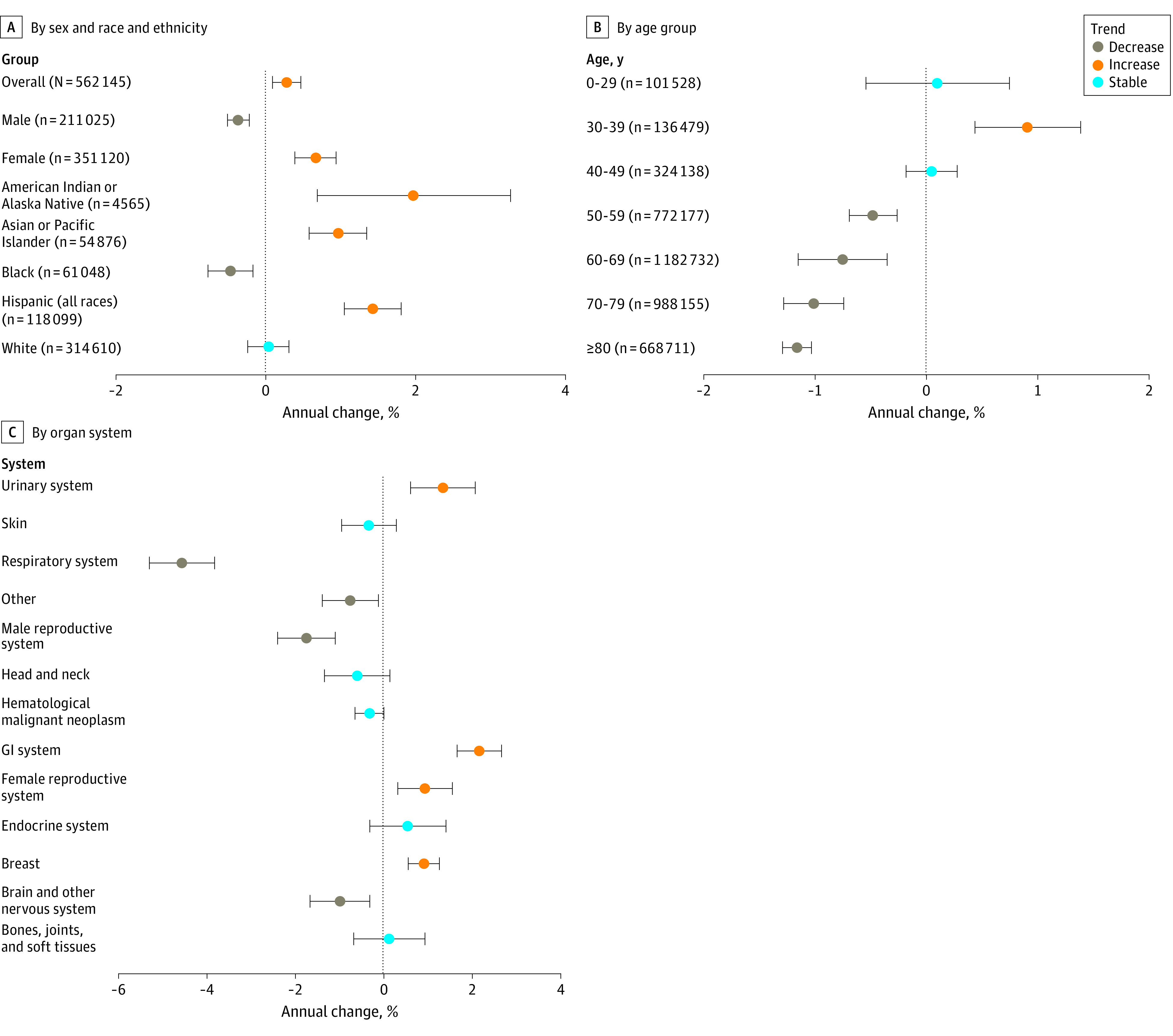
Annual Percentage Change of Early-Onset Cancers From 2010 to 2019 Whiskers indicate 95% CIs. GI indicates gastrointestinal.

The incidence rate of early-onset cancer was 99.96 cases per 100 000 individuals in 2010 and 102.97 cases per 100 000 individuals in 2019. The overall incidence of early-onset cancer increased (APC, 0.28%; 95% CI, 0.09%-0.47%; *P* = .01) (Figure 2A; Table 1). In contrast, the incidence of cancer among individuals 50 years and older decreased over the same period (APC, −0.87%; 95% CI, −1.06% to −0.67%; *P* < .001). During the study period, the age-standardized incidence rates of early-onset cancer increased in female individuals (APC, 0.67%; 95% CI, 0.39%-0.94%; *P* = .001) but decreased in male individuals (APC, −0.37%; 95% CI, −0.51% to −0.22%; *P* < .001) (eFigure 2 in Supplement 1). The mean APCs increased in American Indian or Alaska Native people (1.97%; 95% CI, 0.69%-3.27%; *P* < .001), Asian or Pacific Islander people (0.97%; 95% CI, 0.58%-1.35%; *P* = .007), and Hispanic people (1.43%; 95% CI, 1.05%-1.81%; *P* < .001); were stable in White people (0.04%; 95% CI, −0.24% to 0.31%; *P* = .77); and decreased in Black people (−0.47%; 95% CI, −0.77% to −0.17%; *P* = .007). By age group, the incidence of early-onset cancers increased in individuals aged 30-39 years (APC, 0.91%; 95% CI, 0.44%-1.39%; *P* = .002) but remained stable in other age groups. The incident rates among populations 50 years and older decreased, with mean APCs of −0.48% (95% CI, −0.69% to −0.26%; *P* = .001) among those aged 50 to 59 years, −0.75% (95% CI, −1.15% to −0.35%; *P* = .003) among those aged 60 to 69 years, −1.01% (95% CI, −1.28% to −0.74%; *P* < .001) among those aged 70 to 79 years, and −1.16% (95% CI, −1.29% to −1.03%; *P* < .001) among those 80 years and older (Figure 2B).

**Table 1.** Incidence of Early-Onset Cancers From 2010 to 2019

Characteristic	Incidence (ASIR per 100 000 people)		APC (95% CI)	*P* value^a^
	2010	2019		
Overall	56 051 (99.96)	56 468 (102.97)	0.28 (0.09 to 0.47)	.01
Sex				
Male	21 818 (77.03)	20 747 (74.56)	−0.37 (−0.51 to −0.22)	<.001
Female	34 233 (122.75)	35 721 (131.35)	0.67 (0.39 to 0.94)	.001
Race and ethnicity				
American Indian or Alaska Native^b^	4723 (79.21)	6246 (88.07)	1.97 (0.69 to 3.27)	<.001
Asian or Pacific Islander	351 (101.46)	359 (110.29)	0.97 (0.58 to 1.35)	.007
Black	6245 (85.25)	5953 (93.95)	−0.47 (−0.77 to −0.17)	.007
Hispanic (all races)	10 326 (76.10)	13 177 (87.90)	1.43 (1.05 to 1.81)	<.001
White	33 578 (112.85)	29 481 (113.20)	0.04 (−0.24 to 0.31)	.77
Age group, y				
0-19	4191 (18.32)	3983 (18.08)	0.24 (−0.63 to 1.12)	.54
20-29	5600 (47.72)	5899 (47.39)	−0.01 (−0.55 to 0.53)	.96
30-39	12 361 (113.26)	14 762 (123.83)	0.91 (0.44 to 1.39)	.002
40-49	33 899 (284.44)	31 824 (287.88)	−0.05 (−0.28 to 0.17)	.59

Abbreviations: APC, annual percentage change; ASIR, age-standardized incidence rate.

^a^ *P* value of APC from 2010 to 2019.

^b^ Only data from purchased or referred care delivery areas were included.

### Patterns in the Incidence of Early-Onset Cancer From 2010 to 2019, by Individual Cancers

The incidence rates and APCs of the individual cancers are summarized in Table 2. In 2019, the individual cancers with the highest number of incident early-onset cases were cancers of the breast (n = 12 649), thyroid (n = 5869), and colon and/or rectum (n = 4097). From 2010 to 2019, the cancers with the greatest increase in incident cases were in the appendix (251.89%), intrahepatic bile duct (142.22%), and uterus (76.47%). During the study period, the greatest increase in incidence rates occurred in cancers of the appendix (APC, 15.61%; 95% CI, 9.21%-22.38%; *P* < .001), intrahepatic bile duct (APC, 8.12%; 95% CI, 4.94%-11.39%; *P* < .001), and other digestive organs (APC, 6.63%; 95% CI, 1.58%-11.93%; *P* = .02). The greatest decrease in incidence rates of individual early-onset cancers occurred for cancer in the floor of the mouth (APC, −7.58%; 95% CI, −9.59% to −5.52%; *P* < .001), acute monocytic leukemia (APC, −6.51%; 95% CI, −8.86% to −4.11%; *P* < .001), and prostate cancer (APC, −6.12%; 95% CI, −8.16% to −4.04%; *P* < .001).

**Table 2.** Age-Standardized Incidence Rates per 100 000 Early-Onset Cancers From 2010 to 2019, Stratified by Individual Cancers

Organ system	ASIR per 100 000 early-onset cancers (95% CI)		APC (95% CI)	*P* value^a^
	2010	2019		
All cancers	99.96 (99.14 to 100.80)	102.97 (102.12 to 103.83)	0.28 (0.09 to 0.47)	.01
Bones, soft tissue, and joints				
Bones and joints	0.70 (0.63 to 0.77)	0.82 (0.75 to 0.90)	0.71 (−0.94 to 2.39)	.35
Soft tissue including heart	1.76 (1.65 to 1.87)	1.69 (1.59 to 1.80)	−0.16 (−0.88 to 0.56)	.62
Brain and nervous system				
Brain	3.31 (3.16 to 3.46)	3.00 (0.07 to 2.86)	−0.71 (−1.44 to 0.01)	.20
Cranial nerves and other nervous system	0.38 (0.33 to 0.43)	0.23 (0.20 to 0.28)	−3.47 (−6.29 to −0.55)	.03
Breast				
Breast	21.25 (20.87 to 21.64)	23.74 (23.32 to 24.16)	0.91 (0.55 to 1.26)	<.001
Endocrine				
Thyroid	9.63 (9.38 to 9.90)	10.44 (10.17 to 10.71)	0.55 (−0.36 to 1.46)	.20
Other endocrine including thymus	0.52 (0.46 to 0.58)	0.51 (0.45 to 0.57)	0.36 (−1.45 to 2.21)	.66
Female reproductive				
Cervix uteri	3.01 (2.87 to 3.16)	3.02 (2.88 to 3.17)	0.59 (−0.15 to 1.34)	.10
Corpus uteri	2.77 (2.63 to 2.91)	3.39 (3.24 to 3.55)	2.22 (1.59 to 2.85)	<.001
Ovary	1.94 (1.83 to 2.06)	1.84 (1.73 to 1.96)	−1.03 (−2.15 to 0.10)	.07
Vagina	0.08 (0.06 to 0.11)	0.08 (0.07 to 0.09)	−0.59 (−4.55 to 3.55)	.75
Vulva	0.31 (0.26 to 0.36)	0.27 (0.23 to 0.32)	−0.66 (−2.77 to 1.49)	.49
Other female genital organs	0.16 (0.13 to 0.19)	0.22 (0.18 to 0.27)	6.37 (3.12 to 9.73)	.002
Male reproductive				
Prostate	2.82 (2.69 to 2.96)	1.80 (1.68 to 1.91)	−6.12 (−8.16 to −4.04)	<.001
Testis	3.47 (3.32 to 3.62)	3.67 (3.51 to 3.82)	0.75 (0.16 to 1.35)	.02
Penis	0.08 (0.05 to 0.10)	0.10 (0.07 to 0.13)	1.29 (−1.90 to 4.58)	.38
Other male genital organs	0.03 (0.02 to 0.05)	0.03 (0.02 to 0.05)	−0.70 (−5.39 to 4.21)	.75
Gastrointestinal				
Esophagus	0.40 (0.35 to 0.45)	0.36 (0.31 to 0.42)	−0.94 (−2.56 to 0.71)	.22
Stomach	1.27 (1.18 to 1.37)	1.46 (1.34 to 1.54)	1.59 (0.65 to 2.55)	.005
Small intestine	0.47 (0.42 to 0.53)	0.53 (0.47 to 0.60)	2.11 (0.73 to 3.50)	.008
Cecum	0.60 (0.54 to 0.67)	0.63 (0.57 to 0.70)	0.53 (−0.41 to 1.47)	.23
Appendix	0.33 (0.28 to 0.38)	1.13 (1.05 to 1.23)	15.61 (9.21 to 22.38)	<.001
Colon and/or rectum	6.55 (6.34 to 6.77)	7.63 (7.40 to 7.87)	1.72 (1.23 to 2.21)	<.001
Liver	0.98 (0.90 to 1.07)	0.81 (0.78 to 0.83)	−4.67 (−5.70 to −3.63)	<.001
Intrahepatic bile duct	0.08 (0.06 to 0.10)	0.20 (0.17 to 0.24)	8.12 (4.94 to 11.39)	<.001
Gallbladder	0.10 (0.07 to 0.13)	0.11 (0.09 to 0.15)	2.36 (−1.60 to 6.48)	.21
Other biliary organs	0.17 (0.14 to 0.21)	0.15 (0.12 to 0.19)	−1.08 (−2.85 to 0.74)	.21
Pancreas	1.06 (0.98 to 1.15)	1.30 (1.20 to 1.40)	2.53 (1.69 to 3.38)	<.001
Other digestive organs	0.07 (0.05 to 0.09)	0.12 (0.09 to 0.15)	6.63 (1.58 to 11.93)	.02
Head and neck				
Lip	0.14 (0.11 to 0.18)	0.09 (0.07 to 0.12)	−4.61 (−6.84 to −2.33)	.002
Tongue	0.62 (0.55 to 0.68)	0.65 (0.59 to 0.72)	0.63 (−1.06 to 2.35)	.42
Salivary gland	0.38 (0.33 to 0.44)	0.41 (0.36 to 0.47)	0.75 (−0.63 to 2.16)	.25
Floor of mouth	0.08 (0.06 to 0.10)	0.04 (0.02 to 0.06)	−7.58 (−9.59 to −5.52)	<.001
Gum and other mouth	0.26 (0.22 to 0.30)	0.30 (0.26 to 0.35)	1.16 (−0.42 to 2.77)	.13
Nasopharynx	0.34 (0.30 to 0.40)	0.30 (0.25 to 0.35)	−1.63 (−3.02 to −0.21)	.03
Tonsil	0.52 (0.46 to 0.58)	0.44 (0.39 to 0.50)	−1.67 (−2.58 to −0.75)	.003
Oropharynx	0.07 (0.05 to 0.10)	0.11 (0.09 to 0.14)	5.04 (0.22 to 10.08)	.04
Hypopharynx	0.07 (0.05 to 0.09)	0.05 (0.03 to 0.07)	−4.42 (−7.96 to −0.74)	.02
Other oral cavity and pharynx	0.04 (0.02 to 0.06)	0.01 (0.01 to 0.03)	−5.62 (−14.17 to 3.78)	.20
Nose, nasal cavity, and middle ear	0.20 (0.17 to 0.25)	0.20 (0.19 to 0.21)	−1.44 (−4.12 to 1.32)	.26
Hematological				
Hodgkin–nodal	2.42 (2.29 to 2.55)	2.31 (2.18 to 2.43)	−0.63 (−1.19 to −0.06)	.03
Hodgkin–extranodal	0.03 (0.02 to 0.05)	0.02 (0.01 to 0.04)	−4.06 (−10.80 to 3.20)	.23
NHL–nodal	3.18 (3.03 to 3.33)	2.68 (2.55 to 2.82)	−1.34 (−1.92 to −0.77)	.001
NHL–extranodal	1.73 (1.62 to 1.84)	1.70 (1.59 to 1.81)	−0.05 (−1.14 to 1.05)	.92
Myeloma	0.79 (0.72 to 0.87)	0.83 (0.75 to 0.91)	0.88 (−0.25 to 2.02)	.11
Acute lymphocytic leukemia	1.86 (1.75 to 1.97)	1.97 (1.86 to 2.09)	0.84 (−0.04 to 1.73)	.06
Chronic lymphocytic leukemia	0.36 (0.31 to 0.42)	0.33 (0.29 to 0.39)	−1.52 (−3.08 to 0.07)	.06
Other lymphocytic leukemia	0.11 (0.08 to 0.14)	0.14 (0.11 to 0.18)	0.77 (−1.88 to 3.51)	.52
Acute myeloid leukemia	1.29 (1.20 to 1.39)	1.30 (1.20 to 1.40)	−0.47 (−1.34 to 0.41)	.25
Acute monocytic leukemia	0.11 (0.08 to 0.14)	0.05 (0.04 to 0.08)	−6.51 (−8.86 to −4.11)	<.001
Chronic myeloid leukemia	0.73 (0.66 to 0.80)	0.77 (0.70 to 0.84)	0.76 (−0.67 to 2.22)	.26
Other myeloid or monocytic leukemia	0.05 (0.03 to 0.07)	0.03 (0.02 to 0.05)	−3.53 (−8.97 to 2.23)	.20
Other acute leukemia	0.07 (0.05 to 0.10)	0.10 (0.08 to 0.13)	3.19 (−0.87 to 7.42)	.11
Aleukemic, subleukemic, and NOS	0.10 (0.07 to 0.13)	0.11 (0.10 to 0.12)	1.20 (−1.91 to 4.42)	.41
Respiratory				
Larynx	0.37 (0.32 to 0.42)	0.26 (0.21 to 0.30)	−4.41 (−6.63 to −2.14)	.002
Lung and bronchus	3.48 (3.33 to 3.64)	2.36 (2.23 to 2.50)	−4.72 (−5.50 to −3.93)	.01
Pleura	0.01 (0 to 0.02)	0.01 (0 to 0.02)	NA	NA
Trachea, mediastinum, and other respiratory organs	0.14 (0.11 to 0.17)	0.11 (0.09 to 0.14)	−1.59 (−3.96 to 0.83)	.17
Skin				
Melanoma of the skin	7.50 (7.27 to 7.73)	7.11 (6.89 to 7.34)	−0.36 (−1.01 to 0.28)	.23
Other nonepithelial skin	0.48 (0.42 to 0.54)	0.47 (0.41 to 0.53)	0.10 (−2.06 to 2.32)	.91
Urinary				
Urinary bladder	1.21 (1.12 to 1.31)	1.04 (0.96 to 1.13)	−1.45 (−2.23 to −0.66)	.003
Kidney and renal pelvis	3.61 (3.45 to 3.77)	4.39 (4.22 to 4.58)	2.16 (1.30 to 3.02)	<.001
Ureter	0.02 (0.01 to 0.03)	0.01 (0 to 0.02)	−2.25 (−11.85 to 8.39)	.63
Other urinary organs	0.03 (0.02 to 0.04)	0.02 (0.02 to 0.03)	−0.61 (−7.30 to 6.55)	.85
Other				
Anus, anal canal, and anorectum	0.52 (0.46 to 0.58)	0.45 (0.43 to 0.46)	−3.05 (−4.47 to −1.60)	.001
Retroperitoneum	0.15 (0.12 to 0.19)	0.15 (0.14 to 0.16)	0.33 (−3.81 to 4.65)	.86
Peritoneum, omentum, and mesentery	0.06 (0.04 to 0.08)	0.06 (0.06 to 0.07)	−0.80 (−4.66 to 3.23)	.65
Eye and orbit	0.38 (0.33 to 0.43)	0.31 (0.27 to 0.36)	−1.20 (−2.57 to 0.19)	.08
Kaposi sarcoma	0.43 (0.38 to 0.49)	0.28 (0.24 to 0.33)	−5.01 (−7.68 to −2.26)	.003
Mesothelioma	0.05 (0.04 to 0.08)	0.07 (0.05 to 0.09)	0.98 (−3.40 to 5.57)	.63
Miscellaneous	2.20 (2.08 to 2.33)	2.29 (2.16 to 2.42)	0.42 (−0.24 to 1.08)	.18

Abbreviations: APC, annual percentage change; ASIR, age-standardized incidence rate; NA, not applicable; NHL, non-Hodgkin lymphoma; NOS, not otherwise specified.

^a^ *P* value of APC from 2010 to 2019.

### Patterns in the Incidence of Early-Onset Cancers From 2010 to 2019, by Organ System

The number of incident cases of early-onset cancer in 2010 and 2019 by organ system is shown in Figure 1C and Table 3. In 2019, the highest number of incident cases of early-onset cancer were in cancers of the breast (n = 12 649), gastrointestinal system (n = 7383), and hematological system (n = 6960). From 2010 to 2019, the greatest increases in the number of incident cases of early-onset cancer occurred in the gastrointestinal system (14.80%; from 6431 cases to 7383 cases), breast (7.70%; from 11 745 cases to 12 649 cases), and endocrine system (8.69%; from 5659 cases to 6151 cases). The mean APCs of early-onset cancer from 2010 to 2019 by organ system are shown in Figure 2C and Table 3. From 2010 to 2019, the greatest increases in incidence rates of early-onset cancers occurred in cancers of the gastrointestinal system (APC, 2.16%; 95% CI, 1.66%-2.67%; *P* < .001), urinary system (APC, 1.34%; 95% CI, 0.61%-2.07%; *P* = .003), and female reproductive system (APC, 0.93%; 95% CI, 0.32%-1.55%; *P* = .008) (eFigure 3 in Supplement 1). In contrast, the greatest decreases in incidence rates of early-onset cancers occurred in cancers of the respiratory system (APC, −4.57%; 95% CI, −5.30% to −3.83%; *P* < .001), male reproductive system (APC, −1.75%; 95% CI, −2.40% to −1.10%; *P* < .001), and brain and nervous system (APC, −0.99%; 95% CI, −1.67% to −0.32%; *P* = .01).

**Table 3.** Incidence of Early-Onset Cancers From 2010 to 2019, Stratified by Cancer Site

Organ system	Incidence (ASIR per 100 000 early-onset cancers)		APC (95% CI)	*P* value^a^
	2010	2019		
All cancers	56 051 (99.96)	56 468 (102.97)	0.28 (0.09 to 0.47)	.01
Bones, soft tissue, and joints	1400 (2.46)	1426 (2.52)	0.12 (−0.68 to 0.93)	.74
Brain and nervous system	2097 (3.69)	1821 (3.23)	−0.99 (−1.67 to −0.32)	.01
Breast	11 745 (21.25)	12 649 (23.74)	0.91 (0.55 to 1.26)	<.001
Endocrine	5659 (10.15)	6151 (10.95)	0.54 (−0.32 to 1.41)	.18
Female reproductive	4636 (8.36)	4906 (8.99)	0.93 (0.32 to 1.55)	.008
Male reproductive	3701 (6.40)	3233 (5.59)	−1.75 (−2.40 to −1.10)	<.001
Gastrointestinal	6431 (11.49)	7383 (13.65)	2.16 (1.66 to 2.67)	<.001
Head and neck	1531 (2.72)	1400 (2.57)	−0.60 (−1.34 to 0.14)	.10
Hematological	7266 (12.82)	6960 (12.36)	−0.32 (−0.65 to 0)	.05
Respiratory	2277 (3.99)	1474 (2.74)	−4.57 (−5.30 to −3.83)	<.001
Skin	4451 (7.97)	4161 (7.58)	−0.34 (−0.95 to 0.28)	.25
Urinary	2720 (4.87)	2938 (5.47)	1.34 (0.61 to 2.07)	.003
Other	2137 (3.79)	1966 (3.59)	−0.76 (−1.39 to −0.12)	.06

Abbreviations: APC, annual percentage change; ASIR, age-standardized incidence rate.

^a^ *P* value of APC from 2010 to 2019.

### Patterns in the Incidence of Early-Onset Gastrointestinal Cancers From 2010 to 2019

Because gastrointestinal cancers had the fastest-growing incidence rates among the organ systems, we further explored the incidence patterns among individual gastrointestinal cancers to identify the specific organs contributing to this pattern. The number of incident cases of early-onset gastrointestinal cancers in 2010 and 2019, by individual organ sites, are shown in eFigure 1 in Supplement 1, and the incidence rates and patterns of early-onset gastrointestinal cancers are shown in eTable 3 in Supplement 1. In 2019, the highest number of incident cases of early-onset gastrointestinal cancers occurred in the colon and/or rectum (n = 4097), stomach (n = 773), and pancreas (n = 701). From 2010 to 2019, the greatest increases in the number of incident cases of early-onset cancer were in the appendix (251.89%; from 185 cases to 651 cases), intrahepatic bile duct (142.22%; from 45 cases to 109 cases), and pancreas (18.21%; from 593 cases to 701 cases). The greatest increase in the incidence rates of early-onset gastrointestinal cancers was in the appendix (APC, 15.61%; 95% CI, 9.21%-22.38%; *P* < .001), intrahepatic bile duct (APC, 8.12%; 95% CI, 4.94%-11.39%; *P* < .001), and pancreas (APC, 2.53%; 95% CI, 1.69%-3.38%; *P* < .001) (eTable 3 in Supplement 1). In contrast, the incidence rates of cancers of the liver and esophagus decreased, with APCs of −4.67% (95% CI, −5.70% to −3.63%; *P* < .001) for liver cancer and −0.94% (95% CI, −2.56% to 0.71%; *P* = .22) for esophageal cancer.

### Subgroup and Sensitivity Analyses

The number of incident cases and the incidence rates of early-onset gastrointestinal cancers by sex, age group, and race and ethnicity are shown in eTable 4A to 4C in Supplement 1. Among both male and female individuals, the incidence rates of cancers of the appendix and intrahepatic bile duct increased significantly from 2010 to 2019, while the incidence rates of stomach cancers increased only in female individuals, and the incidence rates of gallbladder cancers increased only in male individuals.

By age, the greatest increase in incidence rates of early-onset gastrointestinal cancers occurred in those aged 30 to 39 years, with significant increases in cancers of the esophagus (APC, 6.86%; 95% CI, 1.77%-12.21%; *P* = .01), small intestine (APC, 4.24%; 95% CI, 1.75%-6.78%; *P* = .004), appendix (APC, 16.20%; 95% CI, 9.81%-22.96%; *P* < .001), pancreas (APC, 4.47%; 95% CI, 1.23%-7.80%; *P* = .01), and intrahepatic bile duct (APC, 8.88%; 95% CI, 2.56%-15.59%; *P* = .01) (eTable 4B and eFigure 4 in Supplement 1).

By race and ethnicity, Hispanic people experienced the greatest increase in incidence rates of gastrointestinal cancers (APC, 3.08%; 95% CI, 2.09%-4.08%; *P* < .001) followed by American Indian or Alaska Native people (APC, 2.83%; 95% CI, 0.51%-5.19%; *P* = .02) and White people (APC, 2.45%; 95% CI, 1.75%-3.15%; *P* < .001) (eTable 4C in Supplement 1). There were no significant increases in early-onset gastrointestinal cancers by specific sites among American Indian or Alaska Native people. Asian or Pacific Islander people experienced significant increases in the incidence of early-onset appendiceal cancers. Hispanic people had significant increases in stomach, appendiceal, colorectal, pancreatic, and intrahepatic bile duct cancers. Among Black people, the incidence of appendiceal and biliary cancer significantly increased. White people experienced significant increases in the incidence of appendiceal and intrahepatic bile duct cancers.

We next evaluated patterns in the APCs of early-onset cancer among 12 cancers identified as related to obesity by the Centers for Disease Control and Prevention^35,36,37^ to assess the association between increasing obesity rates and the incidence of early-onset cancer (eFigure 5 and eTable 5 in Supplement 1). From 2010 to 2019, the APC of obesity-related early-onset cancer was 1.00% (95% CI, 0.69%-1.31%; *P* < .001). The fastest-growing incidence rates of obesity-related cancers were in the pancreas (APC, 2.53%; 95% CI, 1.69%-3.38%; *P* < .001), gallbladder (APC, 2.36%; 95% CI, −1.60% to 6.48%; *P* = .21), and uterus (APC, 2.22%; 95% CI, 1.59%-2.85%; *P* < .001). Conversely, the fastest-decreasing incidence rates among obesity-related cancers were in the liver (APC, 4.67%; 95% CI, −5.70% to −3.63%; *P* < .001), ovary (APC, −1.03%; 95% CI, −2.15% to 0.10%; *P* = .07), and esophagus (APC, −0.94%; 95% CI, −2.56% to 0.71%; *P* = .22).

## Discussion

### Main Findings

In this nationwide cohort study, we found that in 2019, a total of 56 468 early-onset cancers were diagnosed in the US. The overall incidence rate of early-onset cancers increased from 2010 to 2019, while the incidence rate of cancers declined among individuals 50 years and older. During the study period, the incidence rates of early-onset cancers increased in female individuals but declined in male individuals; this increase among female individuals was mainly due to cancers of the uterus and breast. By race and ethnicity, there was an increased incidence of early-onset cancer in American Indian or Alaska Native people, Asian or Pacific Islander people, and Hispanic people. However, the incidence of early-onset cancers remained stable among White people and declined among Black people. By age group, the incidence rate of early-onset cancers increased in individuals aged 30 to 39 years and remained stable in other age groups younger than 50 years.

In 2019, breast cancer had the highest number of incident early-onset cases. By organ system, gastrointestinal cancers had the fastest-growing incidence rates of early-onset cancer, followed by cancers of the urinary system and the female reproductive system. Among gastrointestinal cancers in 2019, the most common types of incident early-onset cancers were in the colon and/or rectum, stomach, and pancreas. During the study period, the gastrointestinal early-onset cancers with the fastest-growing incidence rates were in the appendix, intrahepatic bile duct, and pancreas.

### Findings in the Context of Current Literature

These findings built upon previous US studies that provided data on patterns of early-onset cancer until 2014^24^ and 2015.^27^ These data are also consistent with a recent Global Burden of Disease Study,^3^ which estimated that the highest age-standardized incident rates of early-onset cancer occurred in countries with a high sociodemographic index, such as those in North America. The current study expands on these data by providing granular updated patterns through 2019 by organ system, sex, and race and ethnicity. Several studies^7,22,38,39,40,41,42,43,44,45,46^ have described an increase in specific early-onset cancers, such as those of the colon and/or rectum, breast, kidney, stomach, uterus, endometrium, and pancreas. However, there are limited data that provide a comprehensive updated overview of the latest patterns in early-onset cancer in the US, both overall and by organ system, and the current study fills this knowledge gap.

### Implications for Clinical Practice and Future Research

This nationwide study provides updated evidence that the incidence of early-onset cancers in the US is increasing and highlights several disparities. The increase in early-onset cancer disproportionately occurred among female individuals, American Indian or Alaska Native individuals, Asian or Pacific Islander individuals, and individuals aged 30 to 39 years. Further research is required to fully elucidate the reasons for these disparities. There is a need to inform health care professionals about the increasing incidence of early-onset cancer, and investigations for possible tumors need to be considered when clinically appropriate, even in patients younger than 50 years. These data will be useful for public health specialists and health care policy makers and serve as a call to action for further research into the various environmental factors that may be associated with this concerning pattern.

### Limitations

This study has strengths, including a large sample, data updated through 2019, and detailed subgroup analyses by organ system and individual cancer sites.

The study also has limitations. The generalizability of these findings to populations outside of the US may be unclear. There may have been misclassification, or there could have been underreporting or underdiagnosis among underserved populations, such as Black individuals; hence, these results require cautious interpretation. We recognize that although the Centers for Disease Control and Prevention included ovarian cancer in the group of cancers related to obesity, data regarding the association of body mass index with ovarian cancer are conflicting, with an individual participant data meta-analysis only finding an association between body mass index and ovarian cancer among people who had never used hormonal therapy.^34,47^

## Conclusions

This nationwide cohort study found that the incidence of early-onset cancers continued to increase in the US from 2010 to 2019. While breast cancer had the highest number of incident cases, gastrointestinal cancers had the fastest-growing incidence rates among all early-onset cancers. These data may have implications for the development of surveillance strategies and funding priorities.

## Notes

Supplement 1. **eTable 1.** Categorization of Cancers Within SEER*Stat (Version 8.4.0.1) **eTable 2.** Baseline Characteristics of Early-Onset Cancers From 2010 to 2019^a^ **eTable 3.** Incidence of Early-Onset Gastrointestinal Cancers From 2010 and 2019 **eTable 4A.** Incidence of Early-Onset Gastrointestinal Cancer From 2010 and 2019, Stratified by Sex **eTable 4B.** Incidence of Early-Onset Gastrointestinal Cancer From 2010 and 2019, Stratified by Age Group **eTable 4C.** Incidence of Early-Onset Gastrointestinal Cancer From 2010 and 2019, Stratified by Race **eTable 5.** Incidence of Early-Onset Obesity-Related Cancer From 2010 and 2019 **eFigure 1.** Incident Early-Onset Cancers From 2010 and 2019 **eFigure 2.** Age-Standardized Incidence Rate of Early-Onset Cancers From 2010 to 2019, by Race and Ethnicity, Sex, and Age Group **eFigure 3.** Annual Percentage Change of Cancers From 2010 to 2019, by Organ System **eFigure 4.** Annual Percentage Change of Gastrointestinal Cancers From 2010 to 2019 **eFigure 5.** Annual Percentage Change of Obesity-Related Cancers, by Age Group

Supplement 2.
Data Sharing Statement
