# Supplementary material for: Patterns in Cancer Incidence Among People Younger Than 50 Years in the US, 2010 to 2019
Source: JAMA Netw Open. 2023 Aug 16;6(8):e2328171. doi: 10.1001/jamanetworkopen.2023.28171 (PMC10433086; doi:10.1001/jamanetworkopen.2023.28171)
Supplement: Supplement 1. — eTable 1. Categorization of Cancers Within SEER*Stat (Version 8.4.0.1) eTable 2. Baseline Characteristics of Early-Onset Cancers From 2010 to 2019a eTable 3. Incidence of Early-Onset Gastrointestinal Cancers From 2010 and 2019 eTable 4A. Incidence of Early-Onset Gastrointestinal Cancer From 2010 and 2019, Stratified by Sex eTable 4B. Incidence of Early-Onset Gastrointestinal Cancer From 2010 and 2019, Stratified by Age Group eTable 4C. Incidence of Early-Onset Gastrointestinal Cancer From 2010 and 2019, Stratified by Race eTable 5. Incidence of Early-Onset Obesity-Related Cancer From 2010 and 2019 eFigure 1. Incident Early-Onset Cancers From 2010 and 2019 eFigure 2. Age-Standardized Incidence Rate of Early-Onset Cancers From 2010 to 2019, by Race and Ethnicity, Sex, and Age Group eFigure 3. Annual Percentage Change of Cancers From 2010 to 2019, by Organ System eFigure 4. Annual Percentage Change of Gastrointestinal Cancers From 2010 to 2019 eFigure 5. Annual Percentage Change of Obesity-Related Cancers, by Age Group [file jamanetwopen-e2328171-s001.pdf]

Supplementary Online Content

Koh B, Tan DJH, Ng CH, et al. Patterns in cancer incidence among people younger than 50 years in the US, 2010 to 2019. *JAMA Netw Open*. 2023;6(8):e2328171. doi:10.1001/jamanetworkopen.2023.28171

eTable 1. Categorization of Cancers Within SEER*Stat (Version 8.4.0.1)

eTable 2. Baseline Characteristics of Early-Onset Cancers From 2010 to 2019[a]

eTable 3. Incidence of Early-Onset Gastrointestinal Cancers From 2010 and 2019

eTable 4A. Incidence of Early-Onset Gastrointestinal Cancer From 2010 and 2019, Stratified by Sex

eTable 4B. Incidence of Early-Onset Gastrointestinal Cancer From 2010 and 2019, Stratified by Age Group

eTable 4C. Incidence of Early-Onset Gastrointestinal Cancer From 2010 and 2019, Stratified by Race

eTable 5. Incidence of Early-Onset Obesity-Related Cancer From 2010 and 2019

eFigure 1. Incident Early-Onset Cancers From 2010 and 2019

eFigure 2. Age-Standardized Incidence Rate of Early-Onset Cancers From 2010 to 2019, by Race and Ethnicity, Sex, and Age Group

eFigure 3. Annual Percentage Change of Cancers From 2010 to 2019, by Organ System

eFigure 4. Annual Percentage Change of Gastrointestinal Cancers From 2010 to 2019

eFigure 5. Annual Percentage Change of Obesity-Related Cancers, by Age Group

This supplemental material has been provided by the authors to give readers additional information about their work.

© 2023 Koh B et al. *JAMA Network Open*.

eTable 1. Categorization of Cancers Within SEER*Stat (Version 8.4.0.1)

Category	Includes
**All Cancers**	All
**Head and Neck**	Lip<br>Tongue<br>Salivary Gland<br>Floor of Mouth<br>Gum and Other Mouth<br>Nasopharynx<br>Tonsils<br>Oropharynx<br>Hypopharynx<br>Other Oral Cavity and Pharynx<br>Nose, Nasal Cavity and Middle Ear
**GI (Includes GIT, colorectal and hepatobiliary cancers)**	Oesophagus<br>Stomach<br>Small Intestine<br>Cecum<br>Appendix<br>Ascending Colon<br>Hepatic Flexure<br>Transverse Colon<br>Splenic Flexure<br>Descending Colon<br>Sigmoid Colon<br>Large Intestine, NOS<br>Rectosigmoid Junction<br>Rectum<br>Liver<br>Intrahepatic Bile Duct<br>Gallbladder<br>Other Biliary<br>Pancreas<br>Other Digestive Organs
**Hepatobiliary**	Liver<br>Intrahepatic Bile Duct<br>Gallbladder<br>Other Biliary
**Respiratory System**	Larynx<br>Lung and Bronchus<br>Pleura<br>Trachea, Mediastinum and Other Respiratory Organs
**Bones, Joints, and Soft Tissues**	Bones and Joints<br>Soft Tissues Including Heart
**Skin**	Melanoma of the Skin<br>Other Non-Epithelial Skin
**Breast**	Breast
**Female Genital System**	Cervix Uteri<br>Corpus Uteri<br>Uterus, NOS<br>Ovary<br>Vagina<br>Vulva<br>Other Female Genital Organs
**Male Genital System**	Prostate<br>Testis<br>Penis

© 2023 Koh B et al. *JAMA Network Open*.

Category	Includes
	Other Male Genital Organs
**Urinary System**	Urinary Bladder<br>Kidney and Renal Pelvis<br>Ureter<br>Other Urinary Organs
**Brain and Other Nervous System**	Brain<br>Cranial Nerves Other Nervous System
**Endocrine System**	Thyroid<br>Other Endocrine including Thymus
**Haematological Malignancies**	Hodgkin – Nodal<br>Hodgkin – Extranodal<br>NHL – Nodal<br>NHL – Extranodal<br>Myeloma<br>Acute Lymphocytic Leukaemia<br>Chronic Lymphocytic Leukaemia<br>Acute Myeloid Leukaemia<br>Acute Monocytic Leukaemia<br>Chronic Myeloid Leukaemia<br>Other Myeloid / Monocytic Leukaemia<br>Other Acute Leukaemia<br>Aleukaemic, Subleukaemic and NOS
**Others**	Anus, Anal Canal and Anorectum<br>Retroperitoneum<br>Peritoneum, Omentum and Mesentery<br>Eye and Orbit<br>Kaposi Sarcoma<br>Mesothelioma<br>Miscellaneous
**Individual Cancers**	

© 2023 Koh B et al. *JAMA Network Open*.

eTable 2. Baseline Characteristics of Early-Onset Cancers From 2010 to 2019[a]

	**Age Group, No of Patients (%)**				
	0-19y	20-29y	30-39y	40-49y	Total
**Overall**	42 383 (7.5)	59 145 (10.5)	136 479 (24.3)	324 138 (57.7)	562 145
**Sex**					
Male	22 615 (10.7)	27 175 (12.9)	47 521 (22.5)	113 714 (53.9)	211 025
Female	19 768 (5.6)	31 970 (9.1)	88 958 (25.3)	210 424 (60.0)	351 120
**Race**					
American Indian / Alaska Native[b]	329 (9.1)	413 (11.5)	984 (27.3)	1 879 (52.1)	3 605
Asian / Pacific Islander	3 609 (6.6)	4 877 (8.9)	13 728 (25.0)	32 662 (59.5)	54 876
Black	4 192 (6.9)	5 247 (8.6)	13 880 (22.7)	37 729 (61.8)	61 048
White	20 144 (6.4)	32 372 (10.3)	74 822 (23.8)	187 272 (59.5)	314 610
Hispanic All Races	13 499 (11.4)	14 903 (12.6)	30 238 (25.6)	59 459 (50.3)	118 099
Unknown	559 (6.2)	1 211 (13.5)	2 577 (28.8)	4 600 (51.4)	8 947

[a]Data extracted from the Surveillance, Epidemiology and End Results Database 17 Registry, November 2021 (2000-2019), linked by county
[b]Data only includes PRCDA counties for better representation of American Indian / Alaska Native Population

© 2023 Koh B et al. *JAMA Network Open*.

eTable 3. Incidence of Early-Onset Gastrointestinal Cancers From 2010 and 2019

	2010 Incidence (ASIR per 100,000)	2019 Incidence (ASIR per 100,000)	APC (95% CI)	p[a]
**Overall**	6431 (11.49)	7383 (13.65)	2.16 (1.7 – 2.7)	<0.001
Esophagus	225 (0.40)	196 (0.36)	-0.94 (-2.6 - 0.7)	0.22
Stomach	705 (1.27)	773 (1.44)	1.60 (0.7 – 2.6)	0.005
Small Intestine	261 (0.47)	286 (0.53)	2.11 (0.7 – 3.6)	0.008
Appendix	185 (0.33)	651 (1.13)	15.61 (9.2 – 22.4)	<0.001
Colorectal	3661 (6.55)	4097 (7.63)	1.72 (1.2 – 2.2)	<0.001
Pancreas	593 (1.06)	701 (1.30)	2.53 (1.7 – 3.4)	<0.001
Liver	562 (0.98)	362 (0.66)	-4.67 (-5.7 to -3.6)	<0.001
Gallbladder	57 (0.10)	61 (0.11)	2.36 (-1.6 – 6.5)	0.21
Intrahepatic Bile Duct	45 (0.08)	109 (0.20)	8.12 (4.9 – 11.4)	<0.001
Other Biliary Organs	99 (0.17)	83 (0.15)	-1.08 (-2.9 – 0.7)	0.21
Other Digestive Organs	38 (0.07)	64 (0.12)	6.64 (1.6 – 12.0)	0.02

Abbreviations: ASIR – age-standardized incidence rate; APC – annual percentage change
[a]p-value of APC from 2010 to 2019

© 2023 Koh B et al. *JAMA Network Open*.

eTable 4A. Incidence of Early-Onset Gastrointestinal Cancer From 2010 and 2019, Stratified by Sex

	2010 Incidence (ASIR per 100,000)	2019 Incidence (ASIR per 100,000)	APC (95% CI)	p[a]
**Overall**				
Male	3544 (12.67)	3993 (14.80)	1.77 (1.28 – 2.3)	<0.001
Female	2887 (10.32)	3390 (12.52)	2.64 (1.8 – 3.4)	<0.001
**Esophagus**				
Male	182 (0.65)	151 (0.56)	-1.13 (-3.1 – 0.9)	0.23
Female	43 (0.15)	45 (0.17)	0.01 (-4.6 – 4.8)	0.99
**Stomach**				
Male	379 (1.36)	378 (1.41)	0.36 (-0.6 – 1.3)	0.40
Female	326 (1.18)	395 (1.46)	3.0 (1.6 – 4.4)	0.001
**Small Intestine**				
Male	138 (0.50)	155 (0.59)	2.09 (-0.2 – 4.4)	0.07
Female	123 (0.44)	131 (0.48)	2.08 (-0.4 – 4.7)	0.09
**Appendix**				
Male	77 (0.27)	283 (0.97)	15.58 (10.2 – 21.3)	<0.001
Female	108 (0.38)	368 (1.30)	15.70 (8.4 – 23.4)	0.001
**Colorectal**				
Male	1901 (6.82)	2240 (8.36)	2.21 (1.6 – 2.8)	<0.001
Female	1760 (6.29)	1857 (6.91)	1.15 (0.4 – 1.9)	0.007
**Pancreas**				
Male	326 (1.17)	386 (1.44)	2.06 (0.9 – 3.3)	0.004
Female	267 (0.95)	315 (1.15)	3.13 (1.4 – 4.9)	0.003
**Liver**				
Male	425 (1.49)	249 (0.91)	-5.69 (-6.6 to -4.8)	<0.001
Female	137 (0.48)	113 (0.42)	-1.85 (-4.6 – 1.0)	0.17
**Gallbladder**				
Male	15 (0.05)	20 (0.08)	4.89 (-0.9 – 11.0)	0.09
Female	42 (0.15)	41 (0.15)	1.23 (-2.8 – 5.4)	0.50
**Intrahepatic Bile Duct**				

© 2023 Koh B et al. *JAMA Network Open.*

	2010 Incidence (ASIR per 100,000)	2019 Incidence (ASIR per 100,000)	APC (95% CI)	p[a]
Male	20 (0.07)	57 (0.21)	9.28 (3.9 – 14.9)	0.001
Female	25 (0.09)	52 (0.19)	6.66 (3.6 – 9.8)	0.004
**Other Biliary Organs**				
Male	61 (0.22)	41 (0.15)	-1.68 (-4.5 – 1.3)	0.22
Female	38 (0.13)	42 (0.16)	-0.01 (-3.6 – 3.7)	0.99
**Other Digestive Organs**				
Male	20 (0.07)	33 (0.12)	6.67 (0.8 – 12.9)	0.03
Female	18 (0.06)	31 (0.12)	6.57 (0.6 – 12.9)	0.03

Abbreviations: ASIR – age-standardized incidence rate; APC – annual percentage change
[a]p-value of APC from 2010 to 2019

© 2023 Koh B et al. *JAMA Network Open*.

eTable 4B. Incidence of Early-Onset Gastrointestinal Cancer From 2010 and 2019, Stratified by Age Group

	2010 Incidence (ASIR per 100,000)	2019 Incidence (ASIR per 100,000)	APC (95% CI)	p[a]
**Overall**				
0-19 Years Old	107 (0.46)	224 (1.01)	9.73 (4.6 – 15.1)	0.002
20-29 Years Old	306 (2.60)	485 (3.89)	5.02 (2.7 – 7.4)	0.001
30-39 Years Old	1184 (10.92)	1710 (14.47)	3.46 (2.5 – 4.4)	<0.001
40-49 Years Old	4834 (40.14)	4964 (44.70)	1.32 (0.9 – 1.8)	<0.001
**Esophagus**				
0-19 Years Old	0 (0)	0 (0)	-	-
20-29 Years Old	2 (0.02)	6 (0.5)	1.72 (-11.1 – 16.3)	0.78
30-39 Years Old	21 (0.20)	46 (0.39)	6.86 (1.8 – 12.2)	0.01
40-49 Years Old	202 (1.66)	144 (1.29)	-2.61 (-4.8 to -0.3)	0.03
**Stomach**				
0-19 Years Old	7 (0.03)	3 (0.01)	-1.56 (-11.3 – 9.3)	0.74
20-29 Years Old	41 (0.35)	54 (0.43)	1.52 (-1.8 – 4.9)	0.33
30-39 Years Old	172 (1.59)	203 (1.72)	2.10 (0.2 – 4.1)	0.04
40-49 Years Old	261 (4.06)	286 (4.65)	1.47 (0.2 – 2.7)	0.03
**Small Intestine**				
0-19 Years Old	1 (0.00)	2 (0.01)	-1.31 (-14.1 – 13.4)	0.83
20-29 Years Old	13 (0.11)	12 (0.10)	1.42 (-5.6 – 9.0)	0.66
30-39 Years Old	57 (0.53)	82 (0.70)	4.24 (1.8 – 6.8)	0.004
40-49 Years Old	190 (1.59)	190 (1.72)	1.46 (0.2 – 2.8)	0.03
**Appendix**				
0-19 Years Old	11 (0.05)	109 (0.49)	22.50 (7.2 – 40.1)	0.01
20-29 Years Old	26 (0.22)	165 (1.35)	19.60 (9.4 – 30.7)	0.002
30-39 Years Old	43 (0.39)	179 (1.48)	16.20 (9.8 – 23.0)	<0.001
40-49 Years Old	105 (0.87)	198 (1.81)	10.30 (6.6 – 14.1)	<0.001
**Colorectal**				
0-19 Years Old	12 (0.05)	16 (0.07)	3.62 (-0.4 – 7.8)	0.07
20-29 Years Old	167 (1.42)	175 (1.38)	-0.83 (-1.9 – 0.3)	0.12
30-39 Years Old	699 (6.43)	927 (7.87)	2.06 (1.2 – 2.3)	0.004
40-49 Years Old	2783 (23.15)	2979 (26.81)	1.72 (1.2 – 2.3)	<0.001
**Pancreas**				

© 2023 Koh B et al. *JAMA Network Open*.

	2010 Incidence (ASIR per 100,000)	2019 Incidence (ASIR per 100,000)	APC (95% CI)	p[a]
0-19 Years Old	6 (0.03)	24 (0.11)	16.83 (8.5 – 25.8)	0.001
20-29 Years Old	20 (0.17)	44 (0.35)	10.11 (5.2 – 15.2)	0.001
30-39 Years Old	91 (0.84)	132 (1.11)	4.47 (1.2 – 7.8)	0.01
40-49 Years Old	476 (3.95)	501 (4.49)	1.32 (0.4 – 2.2)	0.01
**Liver**				
0-19 Years Old	67 (0.29)	68 (0.31)	-0.43 (-3.4 – 2.6)	0.75
20-29 Years Old	27 (0.23)	20 (0.16)	-2.97 (-7.9 – 2.2)	0.22
30-39 Years Old	64 (0.60)	65 (0.55)	-0.39 (-3.7 – 1.9)	0.70
40-49 Years Old	404 (3.29)	209 (1.86)	-6.56 (-7.4 to -5.7)	<0.01
**Gallbladder**				
0-19 Years Old	0 (0)	0 (0)	-	-
20-29 Years Old	1 (0.01)	1 (0.01)	-	-
30-39 Years Old	8 (0.08)	9 (0.07)	0.85 (-6.6 – 8.9)	0.81
40-49 Years Old	48 (0.40)	51 (0.46)	2.65 (-1.0 – 6.5)	0.14
**Intrahepatic Bile Duct**				
0-19 Years Old	1 (0.00)	1 (0.00)	-	-
20-29 Years Old	6 (0.05)	4 (0.03)	-	-
30-39 Years Old	7 (0.06)	33 (0.28)	8.88 (2.6 – 15.6)	0.01
40-49 Years Old	31 (0.26)	71 (0.65)	8.01 (4.0 – 12.2)	<0.01
**Other Biliary Organs**				
0-19 Years Old	1 (0.00)	1 (0.00)	-	-
20-29 Years Old	3 (0.03)	2 (0.02)	-1.62 (-9.6 – 7.0)	0.67
30-39 Years Old	11 (0.10)	20 (0.17)	-0.63 (-6.4 – 5.5)	0.81
40-49 Years Old	84 (0.69)	60 (0.54)	-1.37 (-3.5 – 0.9)	0.19
**Other Digestive Organs**				
0-19 Years Old	1 (0.00)	0 (0)	-	-
20-29 Years Old	0 (0)	2 (0.00)	-	-
30-39 Years Old	11 (0.10)	14 (0.12)	3.86 (-5.7 – 14.4)	0.39
40-49 Years Old	26 (0.21)	48 (0.43)	7.25 (1.9 – 12.9)	0.01

Abbreviations: ASIR – age-standardized incidence rate; APC – annual percentage change
[a]p-value of APC from 2010 to 2019

© 2023 Koh B et al. *JAMA Network Open*.

eTable 4C. Incidence of Early-Onset Gastrointestinal Cancer From 2010 and 2019, Stratified by Race

	2010 Incidence (ASIR per 100,000)	2019 Incidence (ASIR per 100,000)	APC (95% CI)	p[a]
**Overall**				
American Indian/ Alaska Native	55 (15.91)	56 (17.92)	2.83 (0.5 – 5.2)	0.02
Asian or Pacific Islander	650 (10.91)	869 (12.27)	0.52 (-0.4 – 1.5)	0.25
Black	925 (14.68)	904 (14.60)	0.71 (-0.2 – 1.6)	0.09
Hispanic	1320 (10.42)	1883 (13.12)	3.08 (2.1 – 4.1)	<0.001
White	3416 (11.18)	3555 (13.68)	2.45 (1.8 – 3.2)	<0.001
**Esophagus**				
American Indian/ Alaska Native	3 (0.83)	2 (0.67)	-0.42 (-8.1 – 7.9)	0.91
Asian or Pacific Islander	15 (0.25)	13 (0.19)	0.10 (-8.4 – 9.4)	0.98
Black	27 (0.42)	21 (0.34)	-0.19 (-5.1 – 5.0)	0.93
Hispanic	33 (0.28)	41 (0.29)	-0.68 (-4.8 – 3.6)	0.72
White	146 (0.46)	118 (0.45)	-0.62 (-2.4 – 1.2)	0.46
**Stomach**				
American Indian/ Alaska Native	12 (3.62)	11 (3.35)	2.82 (-4.9 – 11.2)	0.44
Asian or Pacific Islander	96 (1.60)	99 (1.37)	-0.81 (-2.1 – 0.6)	0.21
Black	94 (1.48)	87 (1.39)	0.10 (-1.9 – 2.1)	0.92
Hispanic	244 (1.90)	346 (2.43)	2.21 (1.3 – 3.2)	0.001
White	255 (0.84)	220 (0.85)	0.65 (-1.0 – 2.4)	0.40
**Small Intestine**				
American Indian/ Alaska Native	3 (0.85)	2 (0.69)	-	-
Asian or Pacific Islander	13 (0.22)	31 (0.45)	4.43 (0.6 – 8.4)	0.03
Black	52 (0.84)	59 (0.95)	1.73 (-1.4 – 5.0)	0.25
Hispanic	35 (0.28)	41 (0.30)	4.40 (-0.5 – 9.6)	0.07
White	155 (0.52)	148 (0.58)	1.61 (-0.4 – 3.7)	0.10
**Appendix**				
American Indian/ Alaska Native	0 (0)	3 (1.05)	-	-
Asian or Pacific Islander	7 (0.12)	43 (0.61)	20.58 (11.9 – 30.0)	<0.001
Black	16 (0.24)	49 (0.75)	14.71 (8.1 – 21.7)	0.001
Hispanic	33 (0.25)	139 (0.85)	20.43 (14.2 – 27.1)	<0.001
White	127 (0.43)	402 (1.50)	17.52 (12.1 – 23.2)	<0.001
**Colorectal**				

© 2023 Koh B et al. *JAMA Network Open*.

	2010 Incidence (ASIR per 100,000)	2019 Incidence (ASIR per 100,000)	APC (95% CI)	p[a]
American Indian/ Alaska Native	28 (8.01)	24 (7.51)	1.42 (-2.6 – 5.7)	0.45
Asian or Pacific Islander	352 (5.92)	482 (6.79)	0.72 (-0.6 – 2.0)	0.23
Black	538 (8.60)	510 (8.29)	0.24 (-0.7 – 1.2)	0.55
Hispanic	623 (4.93)	939 (6.64)	3.50 (2.5 – 4.6)	<0.001
White	2076 (6.81)	2072 (8.00)	1.72 (0.9 – 2.5)	0.001
**Pancreas**				
American Indian/ Alaska Native	1 (0.35)	4 (1.39)	7.90 (0.2 – 16.2)	0.05
Asian or Pacific Islander	36 (0.60)	80 (0.93)	5.10 (0.9 – 9.5)	0.02
Black	100 (1.59)	97 (1.61)	2.51 (-1.9 – 7.1)	0.23
Hispanic	118 (0.95)	173 (1.19)	4.04 (2.0 – 6.2)	0.002
White	336 (1.09)	339 (1.30)	1.69 (0.3 – 3.1)	0.02
**Liver**				
American Indian/ Alaska Native	4 (1.16)	8 (2.70)	0.05 (-5.7 – 6.2)	0.98
Asian or Pacific Islander	101 (1.68)	83 (1.22)	-5.04 (-7.6 to -2.4)	0.002
Black	65 (0.99)	43 (0.65)	-4.44 (-7.3 to -1.5)	0.009
Hispanic	171 (1.32)	99 (0.67)	-7.01 (-9.2 to -4.8)	<0.001
White	215 (0.70)	124 (0.48)	-4.51 (-5.8 to -3.3)	<0.001
**Gallbladder**				
American Indian/ Alaska Native	1 (0.27)	1 (0.24)	-	-
Asian or Pacific Islander	5 (0.09)	9 (0.13)	1.67 (-2.7 – 6.3)	0.86
Black	13 (0.21)	8 (0.13)	-2.85 (-7.0 to 1.5)	0.16
Hispanic	17 (0.14)	28 (0.20)	5.25 (-0.3 – 11.1)	0.06
White	20 (0.06)	15 (0.06)	-0.81 (-10.6 – 10.1)	0.86
**Intrahepatic Bile Duct**				
American Indian/ Alaska Native	0 (0)	0 (0)	-	-
Asian or Pacific Islander	10 (0.17)	15 (0.21)	-1.49 (-13.6 – 12.3)	0.80
Black	4 (0.07)	15 (0.23)	0.38 (-12.7 – 15.4)	0.95
Hispanic	8 (0.06)	33 (0.23)	12.09 (5.6 – 19.0)	0.002
White	23 (0.08)	46 (0.18)	11.14 (6.2 – 16.3)	0.001
**Other Biliary Organs**				
American Indian/ Alaska Native	1 (0.27)	1 (0.33)	-	-
Asian or Pacific Islander	10 (0.17)	10 (0.14)	-3.15 (-6.9 – 0.7)	0.10

© 2023 Koh B et al. *JAMA Network Open*.

	2010 Incidence (ASIR per 100,000)	2019 Incidence (ASIR per 100,000)	APC (95% CI)	p[a]
Black	11 (0.17)	5 (0.08)	-9.08 (-15.4 to -2.3)	0.009
Hispanic	27 (0.22)	26 (0.19)	-0.26 (-3.7 – 3.3)	0.87
White	48 (0.15)	40 (0.16)	-0.85 (-3.0 – 1.4)	0.40
**Other Digestive Organs**				
American Indian/ Alaska Native	2 (0.35)	0 (0)	-	-
Asian or Pacific Islander	5 (0.08)	4 (0.05)	-0.51 (-10.4 – 10.4)	0.91
Black	5 (0.08)	10 (0.17)	23.54 (7.8 – 41.7)	0.007
Hispanic	11 (0.08)	18 (0.13)	2.67 (-1.9 – 7.5)	0.22
White	15 (0.05)	31 (0.12)	10.41 (4.1 – 17.1)	0.005

Abbreviations: ASIR – age-standardized incidence rate; APC – annual percentage change
[a]p-value of APC from 2010 to 2019

© 2023 Koh B et al. *JAMA Network Open*.

eTable 5. Incidence of Early-Onset Obesity-Related Cancer From 2010 and 2019

	2010 Incidence (ASIR per 100,000)	2019 Incidence (ASIR per 100,000)	APC (95% CI)	p[a]
Overall	27 993 (50.36)	30 352 (56.14)	1.00 (0.7 – 1.3)	<0.001
Thyroid	5 362 (9.63)	5 869 (10.44)	0.55 (-0.4 – 1.5)	0.20
Breast	11 745 (21.25)	12 649 (23.74)	0.91 (0.6 – 1.3)	<0.001
Esophagus	225 (0.40)	196 (0.36)	-0.94 (2.6 – 0.7)	0.22
Stomach	705 (1.27)	773 (1.44)	1.60 (0.7 – 2.6)	0.005
Myeloma	439 (0.79)	443 (0.83)	0.88 (-0.3 – 2.0)	0.11
Kidney and Renal Pelvis	2 013 (3.61)	2 359 (4.39)	2.16 (1.3 – 3.0)	<0.001
Corpus Uteri	1 541 (2.77)	1 824 (3.39)	2.22 (1.6 – 2.9)	<0.001
Ovary	1 090 (1.94)	1 018 (1.84)	-1.03 (-2.2 – 0.1)	0.07
Liver	562 (0.98)	362 (0.66)	-4.67 (-5.7 to -3.6)	<0.001
Gallbladder	57 (0.10)	61 (0.11)	2.36 (-1.6 – 6.5)	0.21
Pancreas	593 (1.06)	701 (1.30)	2.53 (1.7 – 3.4)	<0.001
Colorectal	3 661 (6.55)	4 097 (7.63)	1.72 (1.2 – 2.2)	<0.001

Abbreviations: ASIR – age-standardized incidence rate; APC – annual percentage change
[a]p-value of APC from 2010 to 2019

© 2023 Koh B et al. *JAMA Network Open*.

eFigure 1. Incident Early-Onset Cancers From 2010 and 2019

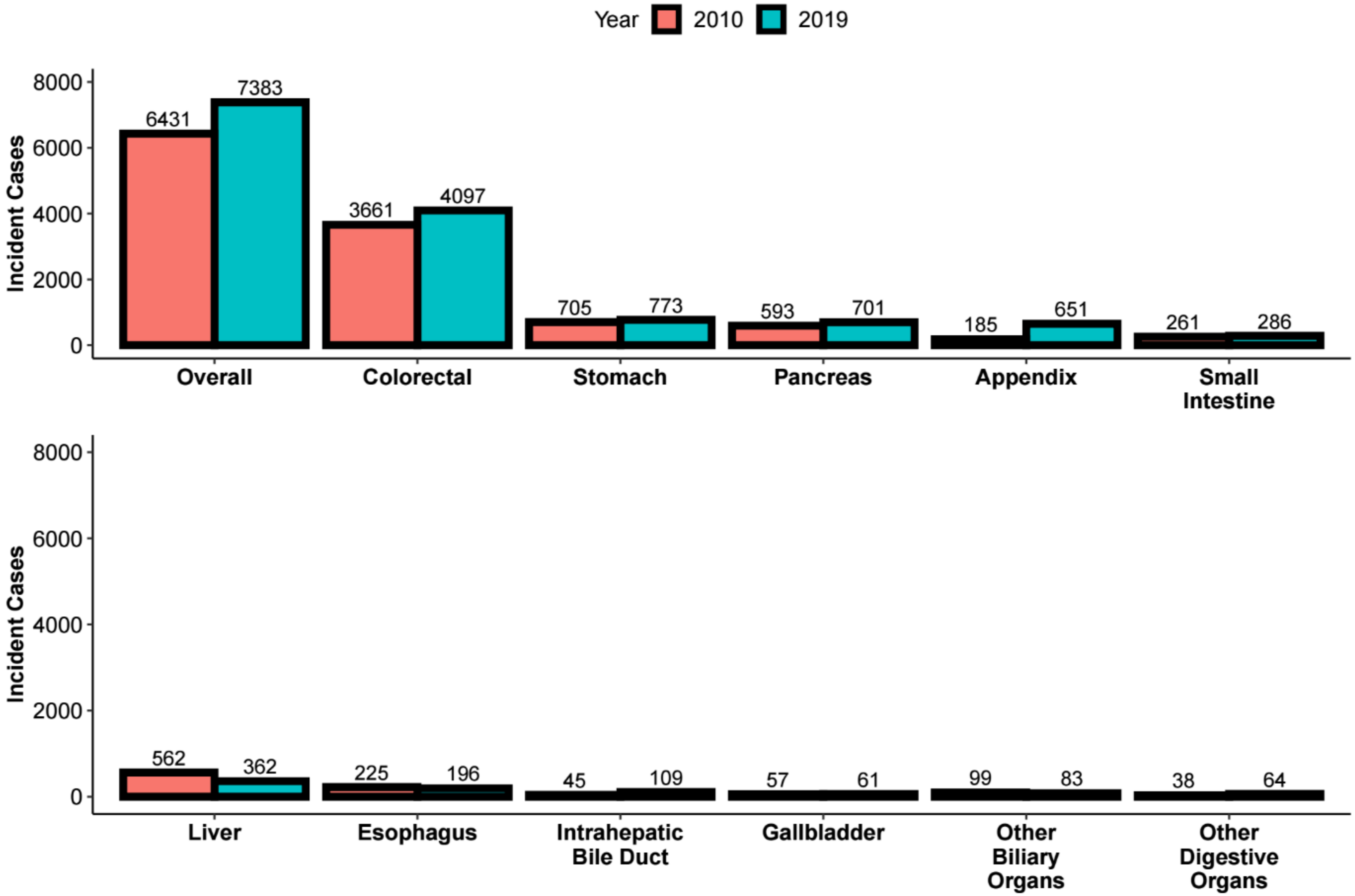

© 2023 Koh B et al. *JAMA Network Open*.

eFigure 2. Age-Standardized Incidence Rate of Early-Onset Cancers From 2010 to 2019, by Race and Ethnicity, Sex, and Age Group

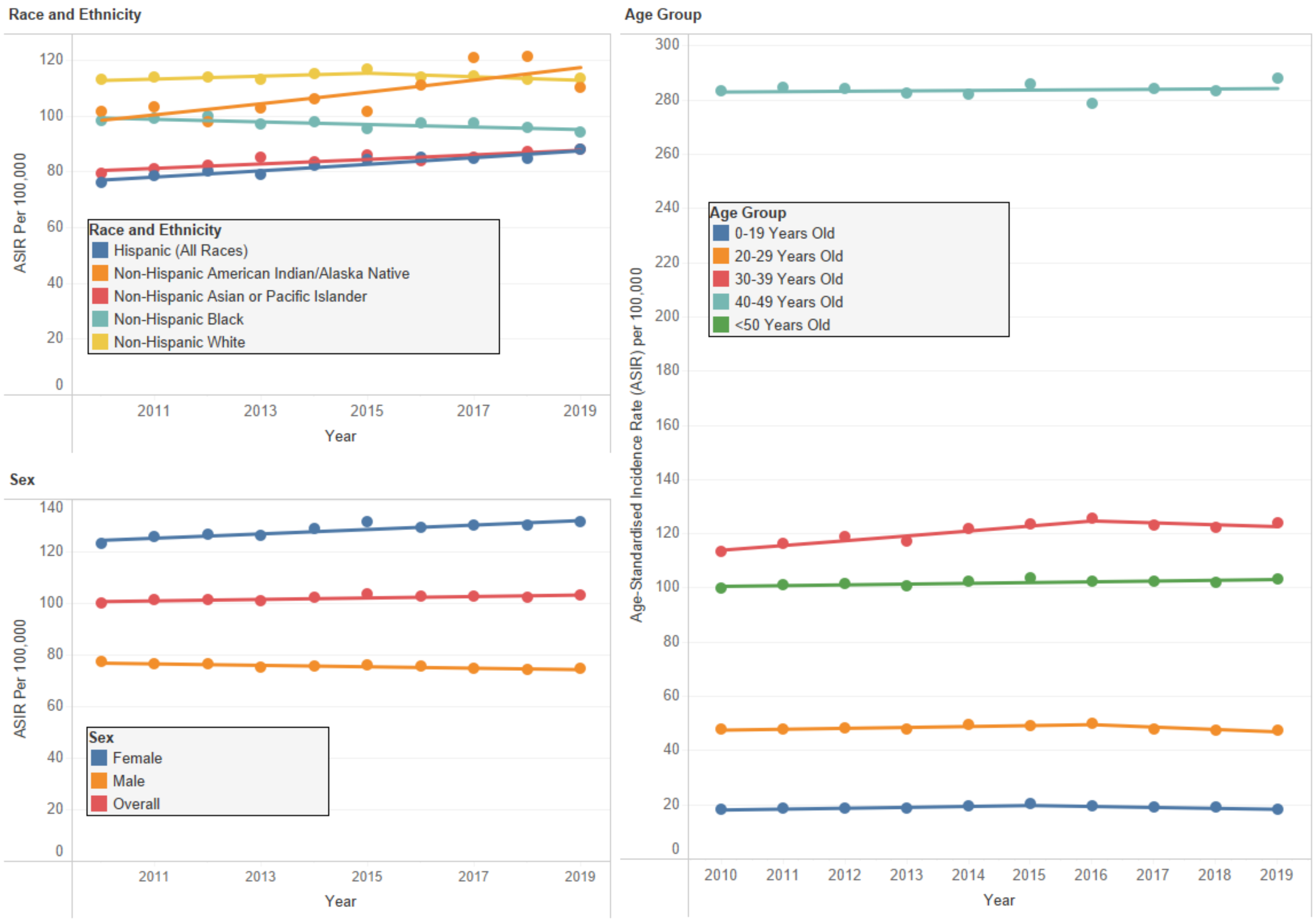

© 2023 Koh B et al. *JAMA Network Open*.

eFigure 3. Annual Percentage Change of Cancers From 2010 to 2019, by Organ System

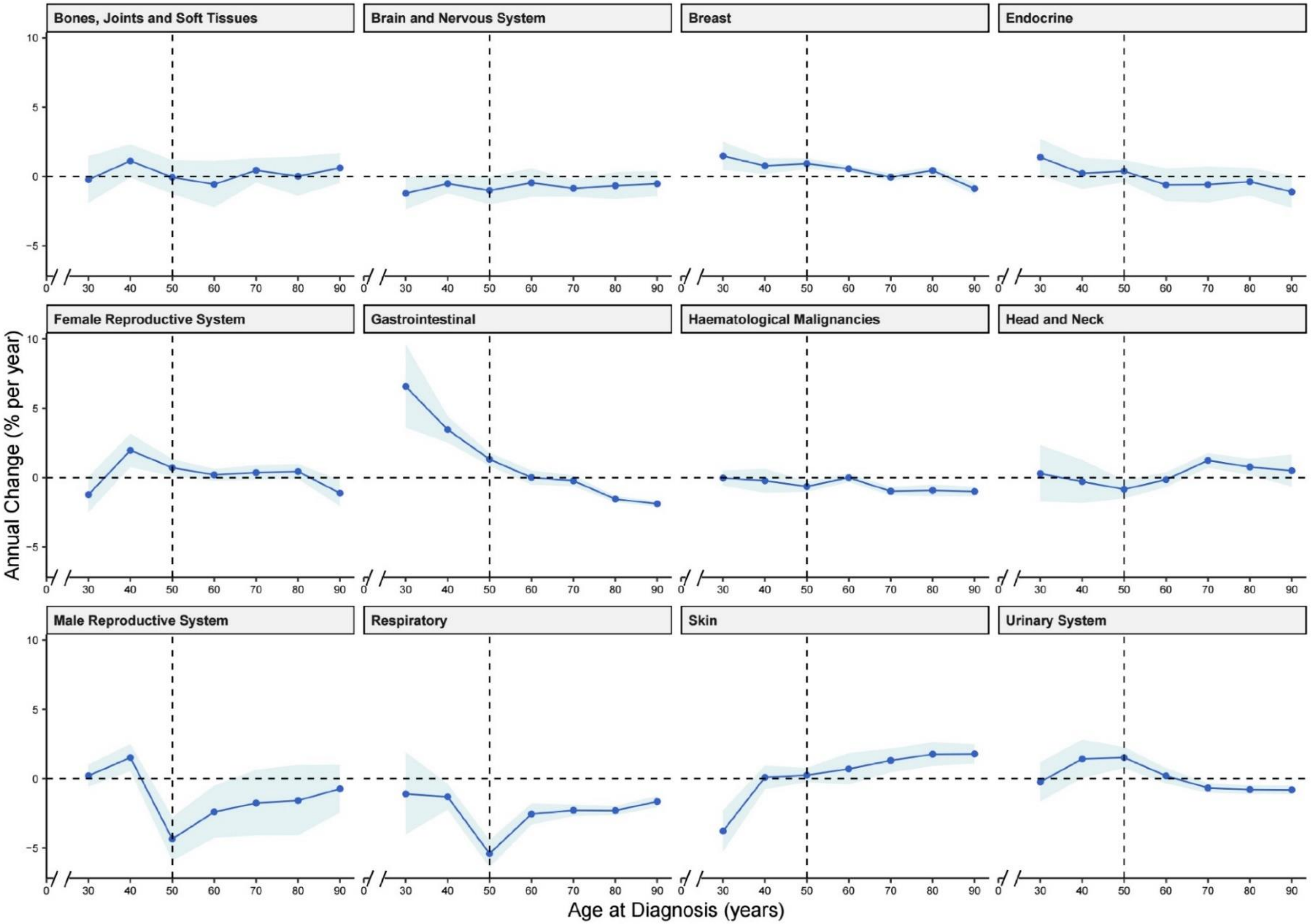

© 2023 Koh B et al. *JAMA Network Open*.

eFigure 4. Annual Percentage Change of Gastrointestinal Cancers From 2010 to 2019

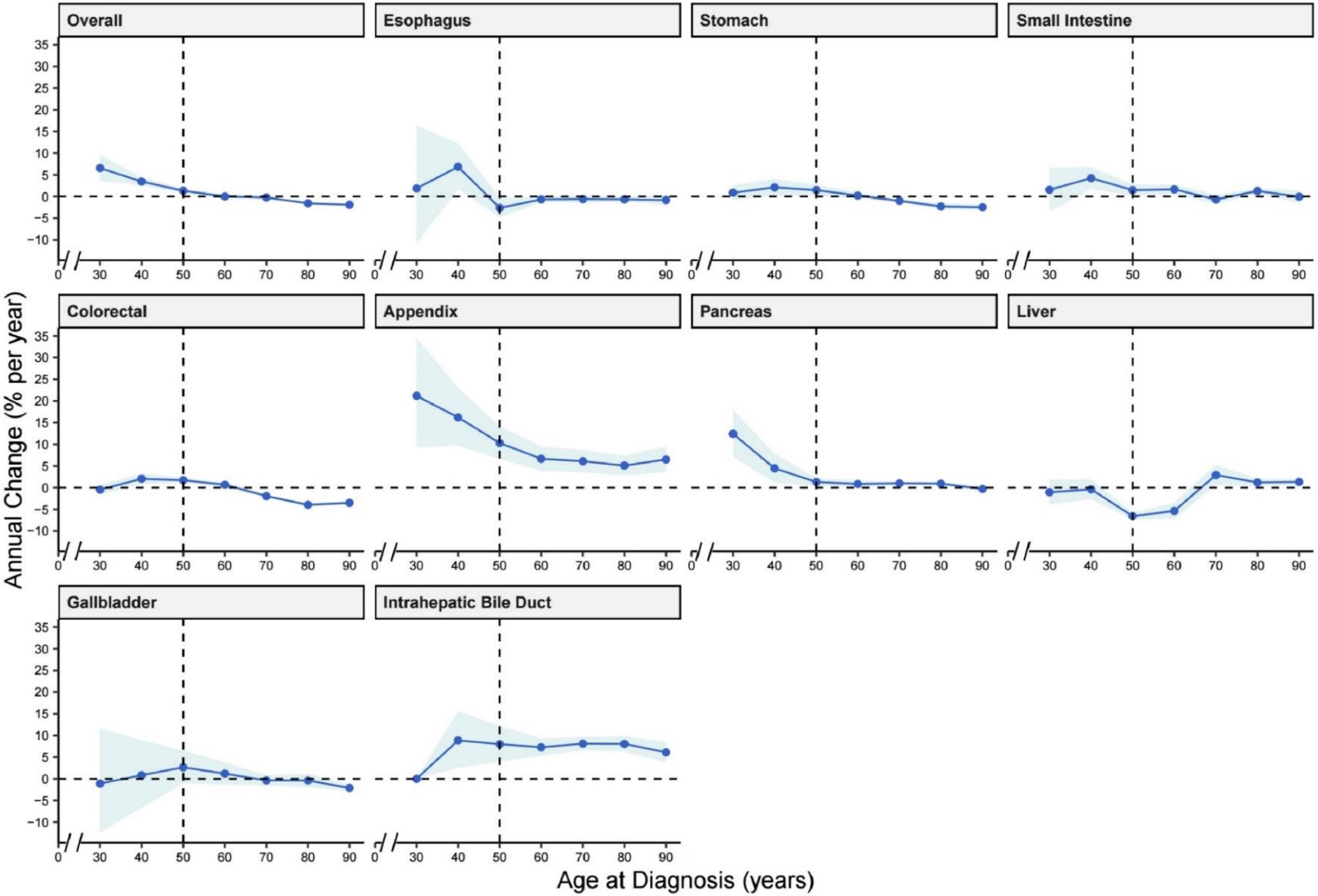

© 2023 Koh B et al. *JAMA Network Open*.

eFigure 5. Annual Percentage Change of Obesity-Related Cancers, by Age Group

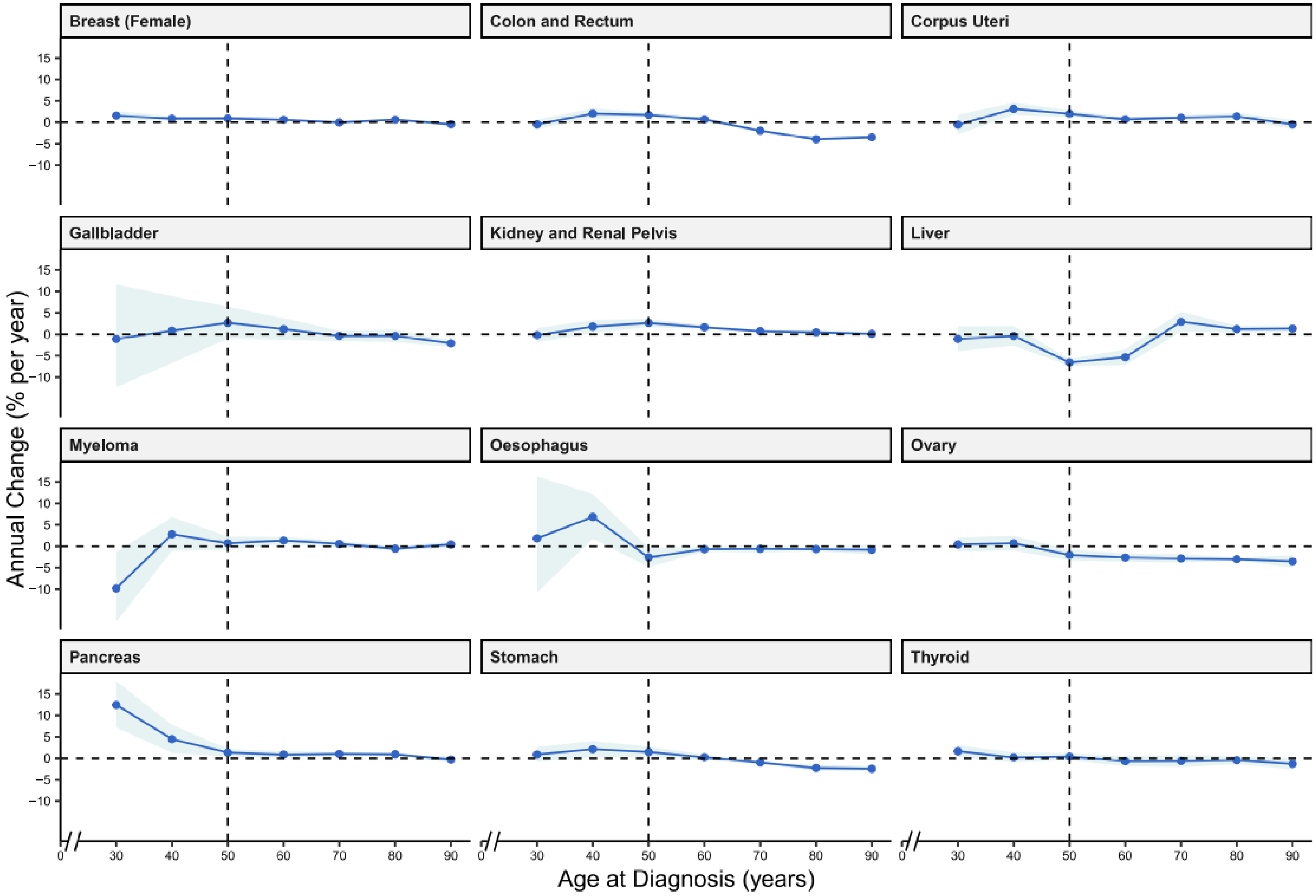

© 2023 Koh B et al. *JAMA Network Open*.
