# Supplementary material for: Patterns in Cancer Incidence Among People Younger Than 50 Years in the US, 2010 to 2019
Source: JAMA Netw Open. 2023 Aug 16;6(8):e2328171. doi: 10.1001/jamanetworkopen.2023.28171 (PMC10433086; doi:10.1001/jamanetworkopen.2023.28171)
Supplement: Supplement 2. — Data Sharing Statement [file jamanetwopen-e2328171-s002.pdf]

Data Sharing Statement

Koh. Patterns in Cancer Incidence Among People Younger Than 50 Years in the US, 2010 to 2019. *JAMA Netw Open*. Published August 16, 2023.
doi:10.1001/jamanetworkopen.2023.28171

Data
Data available: Yes
Data types: Deidentified participant data
How to access data: Surveillance, Epidemiology, and End Results (SEER) 17 registry from 2000 to 2019 https://seer.cancer.gov/data-software/
When available: With publication

Supporting Documents
Document types: None

Additional Information
Who can access the data: Data is an online database accessible via the stated URL
Types of analyses: Data is an online database accessible via the stated URL
Mechanisms of data availability: Data is an online database accessible via the stated URL
